# Dielectric Response and Structural Analysis of (A^3+^, Nb^5+^) Cosubstituted CaCu_3_Ti_4_O_12_ Ceramics (A: Al and Bi)

**DOI:** 10.3390/ma13245822

**Published:** 2020-12-21

**Authors:** Hicham Mahfoz Kotb, Mohamad M. Ahmad, Adil Alshoaibi, Koji Yamada

**Affiliations:** 1 Department of Physics, College of Science, King Faisal University, P.O. Box 400, Al-Ahsa 31982, Saudi Arabia; mmohamad@kfu.edu.sa (M.M.A.); adshoaibi@kfu.edu.sa (A.A.); 2 Physics Department, Faculty of Science, Assiut University, Assiut 71516, Egypt; 3 Department of Physics, Faculty of Science, The New Valley University, El-Kharga 72511, Egypt; 4 Department of Applied Molecular Chemistry, College of Industrial Technology, Nihon University, Narashino, Chiba 275-8575, Japan; yamada.kouji@nihon-u.ac.jp

**Keywords:** milling, sintering, dielectric properties, impedance

## Abstract

CaCu_3_Ti_4-x_((A_0.05_Nb_0.05_))_x_O_12_ ceramics (A: Al and Bi; x = 0, 0.3) were synthesized by high-energy mechanical ball milling and reactive sintering at 1050 °C in air. Rietveld refinement of XRD data revealed the pure and (Al^3+^, Nb^5+^) cosubstituted ceramics contained a minor CuO secondary phase with a mole fraction of about 3.2% and 6.9%, respectively, along with a CaCu_3_Ti_4_O_12_ (CCTO)-like cubic structure. In addition, (Bi^3+^, Nb^5+^) cosubstituted ceramics had a pyrochlore (Ca_2_(Ti, Nb)_2_O_7_) secondary phase of about 18%. While the (Al^3+^, Nb^5+^) cosubstituted CCTO showed the highest relative permittivity (ε’ = 3.9 × 10^4^), pure CCTO showed the lowest dielectric loss (tanδ = 0.023) at 1 kHz and 300 K. Impedance-spectroscopy (IS) measurements showed an electrically heterogeneous structure for the studied ceramics, where a semiconducting grain was surrounded by highly resistive grain boundary. The giant relative permittivity of the ceramics was attributed to the Maxwell–Wagner polarization effect at the blocking grain boundaries and domain boundaries. The higher tanδ of the cosubstituted samples was correlated with their lower grain boundary’s resistivity, as confirmed by IS analysis. Modulus-spectrum analysis revealed two relaxation processes for the pure and (Bi^3+^, Nb^5+^) cosubstituted CCTO samples. Dissimilar behavior was observed for the (Al^3+^, Nb^5+^) cosubstituted CCTO, where three relaxation mechanisms were observed and attributed to the grain, domain-boundary, and grain-boundary responses.

## 1. Introduction

Materials with colossal relative permittivity (ε’ > 10^3^) are important for numerous energy-storage-related applications. In this regard, one of the most promising materials is CaCu_3_Ti_4_O_12_ (CCTO) due to its specific dielectric properties. The relative permittivity of CCTO can attain giant values of 10^4^–10^6^ with little dependency on temperature and frequency of measurement [1]. The origin of the dielectric properties of CCTO is still controversially discussed in the literature. Several studies evidenced an electrically inhomogeneous structure for CCTO and its related materials, i.e., semiconductor grains surrounded by insulating grain boundaries [2]. Considering this polycrystalline structure for CCTO, the model of the internal barrier layer capacitor (IBLC) was proposed by Sinclair et al. [3] and was successfully used in the literature to interpret the dielectric behavior of CCTO. According to this model, the colossal ε’ of CCTO near room temperature and low frequency is due to the extrinsic effect related to Maxwell–Wagner (M–W) polarization at the internal resistive boundaries, such as grain boundaries and/or domain boundaries [4,5]. At a high frequency of the applied electric field, charge accumulation at the internal boundaries was reduced; hence, a decrease in ε′ was observed. An additional extrinsic effect from the sample/electrode interface was also suggested to contribute to the colossal permittivity of CCTO and its related materials [3]. Nevertheless, the use of CCTO as a dielectric material in technology is hindered by its high dielectric loss (tanδ > 0.05). Therefore, research continues for better understanding and improving the dielectric response of CCTO, and to suggest alternative colossal permittivity dielectric materials. In this regard, several strategies are being implemented, such as doping, substitution, and controlling synthesis conditions [6,7,8,9,10,11,12,13,14,15,16,17]. In particular, cosubstitution for Ti^4+^ with heterovalent elements was reported as a promising technique to reduce tanδ without degrading the ε’ of TiO_2_ and CCTO ceramics [12,18,19,20,21]. For instance, (Al^3+^, Nb^5+^) cosubstituents resulted in the increased relative permittivity (ε’ ≈ 2.9−4.1 × 10^4^) and decreased dielectric loss (tanδ ≈ 0.045–0.058) of CCTO ceramics [12]. Ceramics in [12] were prepared by a solid-state reaction process comprising a calcination step at 850 °C for 12 h and conventional sintering at 1050–1090 °C for 3–36 h. Several mechanisms were proposed to explain the colossal relative permittivity of the cosubstituted CCTO, including the confinement of charge-carrier hopping by extrinsic defect clusters [20,22] and the formation of internal capacitors at the internal boundaries of the ceramic (IBLC model) [12]. According to the IBLC model, the colossal permittivity of the ceramics is a result of the internal capacitances that form due to the accumulation of charge carriers at the internal resistive boundaries of the tested sample [3]. Considering this structure, the static relative permittivity $\varepsilon_{s}^{\prime }$ of IBLC ceramics depends on the thickness of the grain boundary (*t_g.b._*), the average grain size (*t_g_*), and the relative permittivity of the grain boundary (*ε_r_*), as follows [23,24]:

(1) $$\varepsilon_{s}^{\prime }=\frac{t_{g}}{t_{g.b.}}\varepsilon_{r}^{\prime }$$

In the present work, we investigated the structural and dielectric properties of cosubstituted CCTO with the composition of CaCu_3_Ti_4−x_[(A_0.05_Nb_0.05_)]_x_O_12_ (A: Al and Bi; x = 0, 0.3). The investigated ceramics were prepared by a simple reactive solid-state reaction process where the calcination step was dismissed. The reactive sintering process has the advantages of having lower thermal budget and more control on the grain size of the final ceramics [14]. To the best of our knowledge, there are no previous reports on the dielectric properties of (Bi^3+^, Nb^5+^) or (Al^3+^, Nb^5+^)-cosubstituted CCTO ceramics prepared by reactive sintering. Few reports exist on (Al^3+^, Nb^5+^)-cosubstituted CCTO ceramics prepared by conventional solid-state reaction [12]. The structural and microstructural properties of the prepared ceramics were studied using X-ray diffraction and FE-SEM measurements. The dielectric properties of the prepared ceramics were studied in a wide range of frequencies (1 Hz–10 MHz) and temperatures (120–400 K).

## 2. Materials and Methods

Powders of CaCu_3_Ti_4−x_((A_0.05_Nb_0.05_))_x_O_12_ (A: Al and Bi; x = 0, 0.3) were synthesized using mechanochemical milling (Fritsch P-7 premium line machine). Stoichiometric amounts of high-purity CaCO_3_, CuO, TiO_2_, Nb_2_O_5_, Bi_2_O_3_, and Al_2_O_3_ were ball-milled with 2-propanol as the medium for 20 h at a rotation speed of 600 rpm. The mass ratio of grinding balls to powder was 8:1. After drying the mixture at 200 °C for 12 h, about 0.5 g of the obtained powder was pressed at pressure of 200 MPa for 3 min using a uniaxial hydraulic press. The obtained green pellet was then sintered in air at 1050 °C for 15 h. Prepared samples are referred to as CCTO, CCTANO, and CCTBNO. Field-emission scanning electron microscope (FE-SEM) (Joel, SM7600F, Tokyo, Japan) was used to study the microstructure of the ceramics. X-ray diffraction (XRD) measurements in the range of 10° ≤ 2θ ≤ 90° were collected using a Bruker D8 Advance X-ray powder diffractometer (CuKα radiation, Karlsruhe, Germany). A turnkey concept 50 system from Novocontrol was used for impedance-spectroscopy (IS) measurements over the 1 Hz–10 MHz frequency range and 120–400 K temperature range in a dry nitrogen atmosphere. Sputtered gold electrodes were used for electrical measurements.

## 3. Results

**Figure 1:**
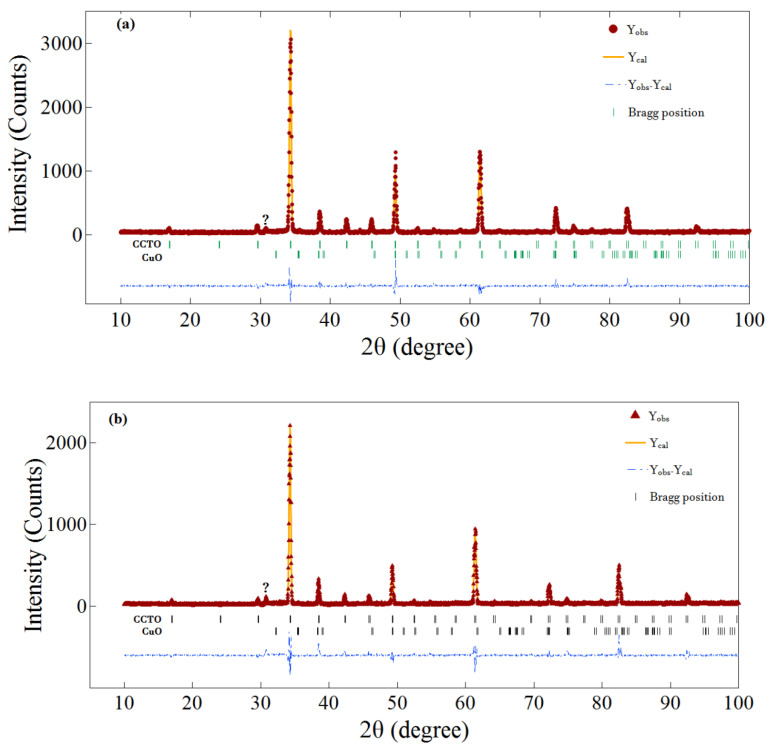

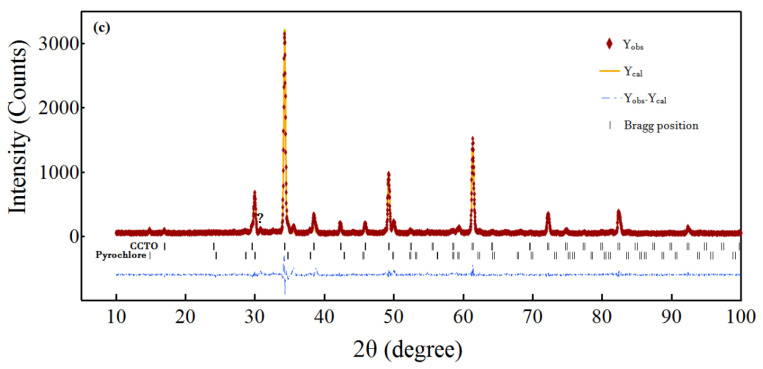
Rietveld refinements of XRD patterns of (**a**) CaCu_3_Ti_4_O_12_ (CCTO), (**b**) CCTANO, and (**c**) CCTBNO ceramics. Y_obs_, experimental data; Y_cal_, calculated data. The symbol (?) represents an unindexed peak.

The X-ray diffraction pattern of the powder (not shown here) showed the onset of formation of a CCTO-like cubic phase during the mechanical milling step. The diffraction patterns of the ceramic samples are shown in Figure 1. Though the majority of diffraction peaks for all ceramics could be indexed as a CCTO cubic phase (JCPDS file no. 75–2188), additional peaks of other secondary phases were observed. Therefore, the Rietveld refinement method was deployed using RIETAN-2000 software [25] to elucidate the structural parameters, and to identify and determine the percentage of the secondary phase. The refined structure parameters and agreement factors of the profile are shown in Table 1. The pure and CCTANO ceramics contained a minor CuO secondary phase with a mole fraction of about 3.2% and 6.9%, respectively. CCTBNO showed a considerable pyrochlore (Ca_2_(Ti, Nb)_2_O_7_) secondary phase of about 18%. Moreover, CCTBNO ceramics showed an increased lattice parameter compared to that of CCTO due to the larger ionic radius of dopants Nb^5+^ (69 pm) and Bi^3+^ (96 pm) compared to Ti^4+^ (61 pm). Additionally, a peak at 2θ = 30.56° was observed for all samples that could not be univocally indexed. 

**Figure 2:**
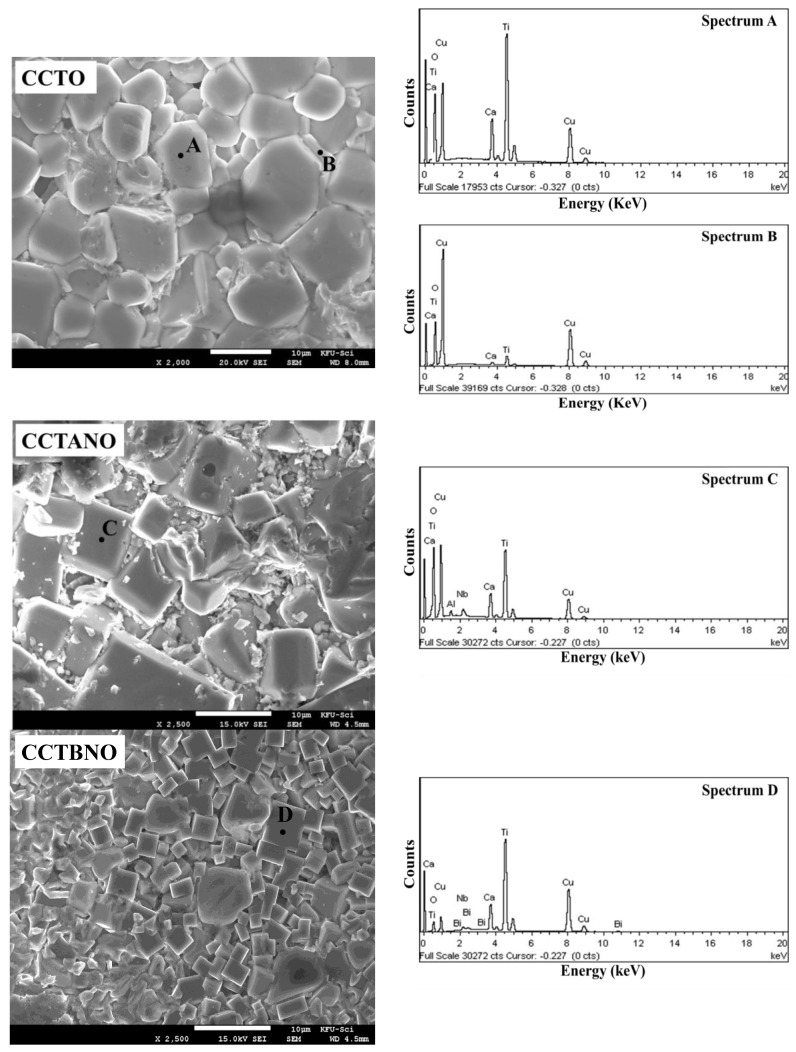
(**left**) FE-SEM micrographs for fractured surface of CCTO, CCTANO, and CCTBNO ceramic samples; (**right**) EDX spectrum for marked points.

Figure 2 depicts the microstructure of CCTO, CCTANO, and CCTBNO ceramics as studied by FE-SEM. Similar grain size distribution was observed for CCTO (7–9 μm) and CCTANO (6–12 μm), while CCTBNO demonstrated a rather smaller but uniform grain size of ~3 μm. These grain-size values were clearly smaller than those in the literature for pure and cosubstituted CCTO ceramics prepared by a conventional solid state reaction (SSR) technique, which is in the 20–40 μm range [12,26,27]. The effect of doping on the grain size of CCTO ceramics was previously studied, where a reduction in the grain size of CCTO ceramics was observed that was attributed to the solute drag effect of the dopants [28,29]. Therefore, the smaller grain size of CCTBNO compared to that of other samples of the present study suggests that Bi dopants have more dragging force than that of Al.

Elemental analysis was carried out using energy dispersive X-ray (EDX) as shown in Figure 2. The elements of each composition were detected, and found to be uniformly distributed across grains and grain boundaries. Element-mole ratios obtained from EDX analysis of the grain were Ca:Cu:Ti = 1:2.87:3.97, Ca:Cu:Ti:Nb:Al = 1:3:3.68:0.16:0.17, and Ca:Cu:Ti:Nb:Bi = 1:3:3.68:0.17:0.13 for the CCTO, CCTANO, and CCTBNO ceramics, respectively. These results were close to the expected theoretical values based on the stoichiometry of each composition. Nevertheless, grains of the CCTO sample were found to be frequently surrounded by Cu-rich regions, as shown in Point B in Figure 2, where the element-mole ratio was found to be Ca:Cu:Ti = 1:42.91:3.92. These Cu-rich regions were probably due to the formation of a liquid CuO phase that wet the grains during sintering. 

**Figure 3:**
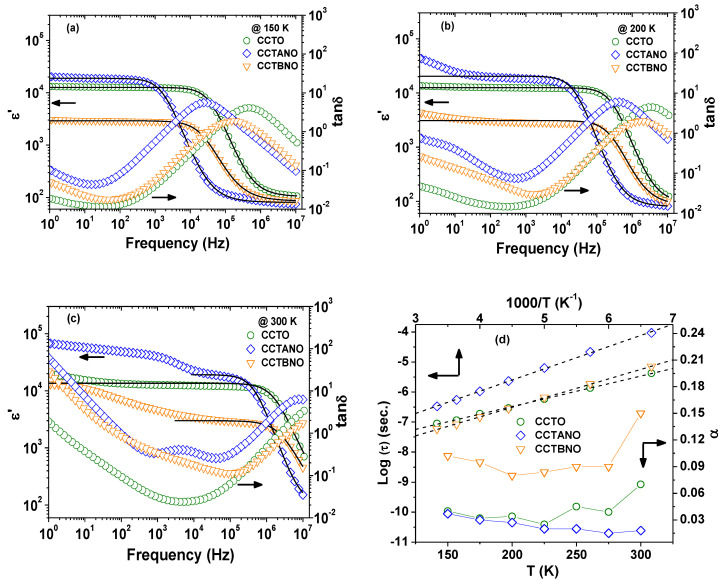
Spectra of *ε’* and tanδ for CCTO, CCTANO, and CCTBNO ceramics at (**a**) 150, (**b**) 200, and (**c**) 300 K; (**d**) temperature dependence of *τ* and *α*. Solid and dashed lines, fitting results by modified Debye equation and Arrhenius relation, respectively.

Figure 3a–c present the spectra of relative permittivity ε’ and dielectric loss tanδ at the selected temperatures of 150, 200, and 300 K. The frequency dependency of ε’ at 150 K for all samples showed one plateau followed by a steplike decrease in the high-frequency range, which is accompanied by a peak in the spectra of tanδ. With increasing temperature, ε’ increased, and the peak of tanδ shifted towards higher frequencies. This dielectric behavior was similar to a Debye-like relaxation process [4,30], which is generally related to the dipole relaxation in the system; however, it sometimes originates from the electrical heterogeneity of the system [31]. As shown by the solid lines in Figure 3a–c, the high-frequency relative permittivity spectra fit well with the modified Debye equation [32]:

(2) $$\varepsilon^{*}=\varepsilon^{\prime }-i\varepsilon^{\prime \prime }=\varepsilon_{\infty }+\left(\varepsilon_{s}-\varepsilon_{\infty }\right)/\left[1+{\left(iwt\right)}^{1-\propto }\right],$$

where *ε_s_* is static relative permittivity, *ε*_∞_ is the relative permittivity at high frequency, *ω* is angular frequency, *τ* is relaxation time, and *α* is a measure of the distribution of relaxation time (0 < *α* ≤ 1). For an ideal Debye relaxation, *α* = 1. Nevertheless, deviation from the modified Debye relaxation was observed for all ceramics at a low frequency, which denoted that there was an additional source of polarization responsible for the giant relative permittivity of the current ceramics. The temperature dependence of the extracted fitting parameters (τ and *α*) is given in Figure 3d. Values of *α* were found to be in the range of 0.02–0.15 for all samples, which indicated a distribution of relaxation time. Additionally, the temperature dependence of the fitted τ values was found to follow the Arrhenius law [33]:

(3) $$\tau =\tau_{0}\;\exp\;\left(\frac{E_{R}}{k_{B}T}\right),$$

where *τ*_0_ and *E_R_* are the pre-exponential factor and the activation energy for the relaxation, respectively. The calculated values of *E_R_* were 0.1, 0.125, and 0.147 eV for CCTO, CCTANO, and CCTBNO, respectively. These *E_R_* values were found to be similar to the relaxation energy in grain calculated from the analysis of modulus spectra of the samples, as is elaborated later in this section. Moreover, Figure 3 shows that pure CCTO displayed a superior dielectric property in terms of better *ε’* stability and lower tanδ values over the studied frequency and temperature ranges. The CCTANO sample exhibited considerably superior *ε’* values in the frequency range (1–10^6^ Hz) at room temperature. The values of *ε’* at 1 kHz and 300 K were 1.2 × 10^4^, 3.9 × 10^4^, and 4.6 × 10^3^ for the CCTO, CCTANO, and CCTBNO ceramics, respectively.

The values of minimal tanδ at room temperature were 0.023 (at 1 kHz), 0.255 (at 40 kHz), and 0.114 (at 115 kHz) for the CCTO, CCTANO, and CCTBNO ceramics, respectively. The dielectric performance of the CCTANO sample was comparable to that reported for CaCu_3_Ti_4−x_[(Al_0.05_Nb_0.05_)]_x_O_12_ (x:0–0.2) prepared by a high-temperature SSR method (*ε’* = 2.9−4.1 × 10^4^, tanδ = 0.045–0.058 at 1 kHz) [12]. 

**Figure 4:**
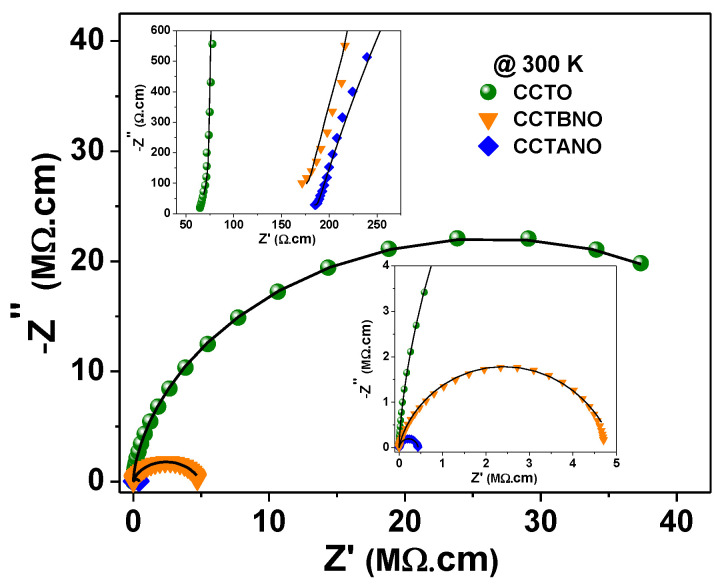
Room-temperature complex-impedance plan (Z*) plots for CCTO, CCTBNO, and CCTANO ceramics. Solid lines, fitting of measured data to equivalent circuits (Table 2).

**Figure 5:**
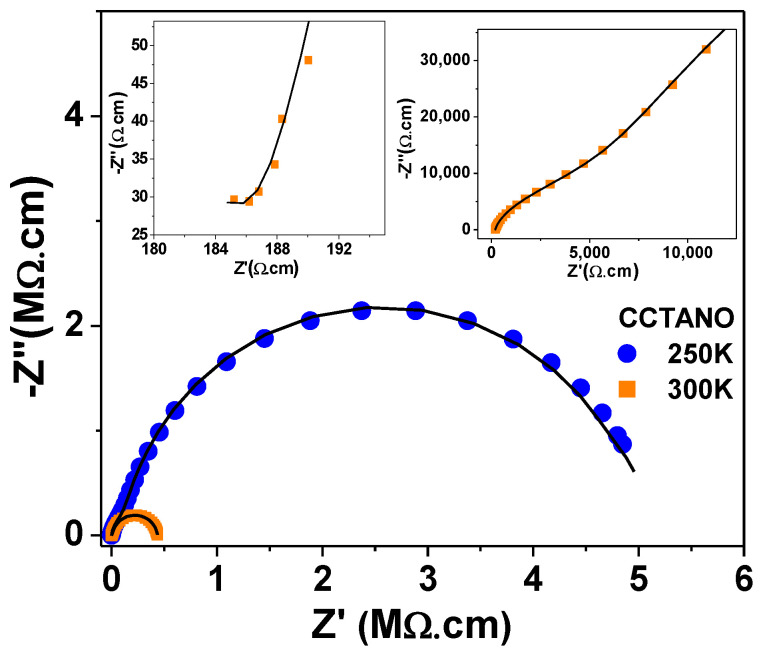
Complex-impedance plan (Z*) plots for CCTO, CCTANO, and CCTBNO ceramics at selected temperatures. Solid lines, fitting of measured data to equivalent circuit (Table 2).

Figure 2 and Figure 3 show that CCTBNO had the lowest *ε’* and smallest grain size. The CCTANO sample had a slightly larger grain size than that of CCTO, which correlated with the larger *ε’* of the former. Therefore, the dielectric behavior of the CCTO, CCTANO, and CCTBNO ceramic samples tended to follow the IBLC model, where relative permittivity is correlated with grain size. Moreover, the lower dielectric performance of the CCTBNO ceramic was thought to be due to its lower CCTO-like phase content, as revealed by the Rietveld refinement analysis. Figure 4 shows the complex-impedance (Z*) plots at room temperature for the pure and cosubstituted CCTO ceramics of the current study. On the one hand, CCTO and CCTBNO samples showed similar features for Z* plot at room temperature, where a large semicircular arc that spread over a wide range of frequencies was observed. Nevertheless, the expanded view of the high-frequency region (see inset) shows a nonzero intercept of the impedance spectra. It is broadly accepted that the nonzero intercept at a high frequency is attributed to the grain response, whereas the large arc at a low frequency is assigned to the grain-boundary response [23]. Resistivity values for the grain and grain boundary, estimated from the intercept of the semicircular arcs with the Z’ axis, were found to be 70 and ~50 MΩ.cm for CCTO, and 187 and 4.7 MΩ.cm for CCTBNO. On the other hand, careful inspection of the Z* plot for CCTANO revealed the existence of three semicircular arcs at high, medium, and low frequencies, respectively, as shown in Figure 5 and the inserted expanded views. Thus, besides the grain response, additional contributions from grain boundaries and domain boundaries, and/or the electrode effect were possibly active in the CCTANO ceramics. The estimated resistivity values from the three arcs at room temperature were 187 Ω.cm (high frequency), 4.51 kΩ.cm (medium frequency), and 0.43 MΩ.cm (low frequency).

Moreover, Figure 4 and Figure 5 show that the semicircular arcs of the complex-impedance plots were depressed, which confirmed the nonideal Debye behavior as previously discussed in Figure 3. To model a nonideal Debye response, a constant phase element (CPE) is often used in the model equivalent circuit [34,35]. The capacitance value of the CPE_i_ element is calculated using the following relation [36,37]:

$$C_{i}=\frac{{\left[R_{i}.Q_{i}\right]}^{\frac{1}{n_{i}}}}{R_{i}}$$

where *R_i_*, *Q_i_*, and *n_i_* are the CPE_i_’s fitting parameters, and *n* ranges from *n* = 0 for the purely resistant behavior to *n* = 1 for the purely capacitive behavior of the CPE element. Experimental complex-impedance data were well-fitted to the equivalent circuits shown in Table 2 using ZSimpWin (v3.10, Ametek, EChem software, Ann Arbor, MI, USA). In all cases, the chi^2^ value was in the order of 10^−3^–10^−4^, which reflects the goodness of fit as shown by the solid lines in Figure 4 and Figure 5. The obtained fitting parameters for the experimental data at 200 and 300 K are summarized in Table 2. The values of n were found to lie in the range of 0.91–0.98, indicating the close proximity of capacitance behavior. The estimated resistivity of the grain, grain boundary, and domain boundary (R_g_) was close to their values calculated by the intercept method.

**Figure 6:**
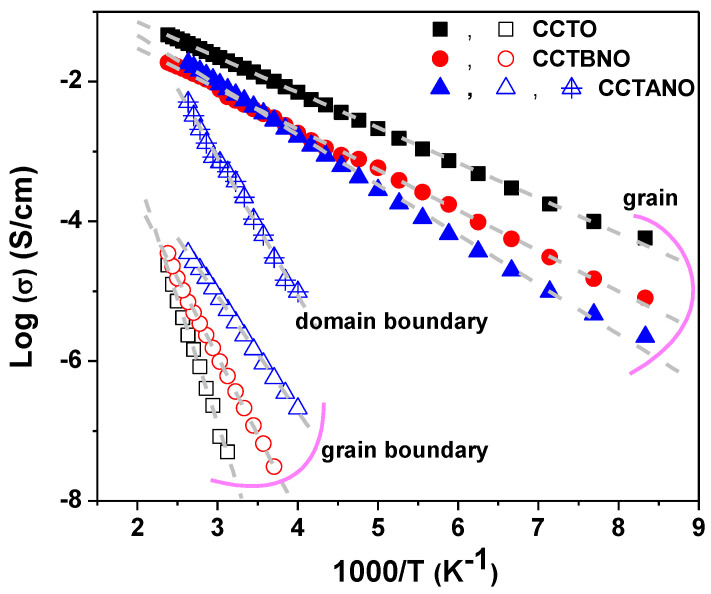
Arrhenius plots of conductivity for CCTO, CCTBNO, and CCTANO ceramics.

Thus, complex-impedance measurements of the current samples revealed their electrical inhomogeneity, where they were composed of semiconductor grains and electrically insulating regions in the form of domain and/or grain boundaries. This electrically inhomogeneous structure suggested the Maxwell–Wagner (M–W) polarization effect as the origin of the giant relative permittivity of the current samples, as shown in Figure 3. M–W polarization ascribes the effect of the polarization that takes place at blocking interfaces and boundaries such as sample surface/electrode, grain/grain boundary, and domain/domain boundary [4,5]. Figure 6 depicts the Arrhenius plots for the conductivity of the grain, domain-boundary, and grain-boundary regions. The activation-energy values for conduction could be calculated using the Arrhenius relation [33]:

(4) $$\sigma =\;\sigma_{0}\;\exp\;\left(\frac{-E_{a}}{k_{B}T}\right)$$

where *σ*_0_ is the pre-exponential factor, *k_B_* is Boltzmann constant, and *E_a_* is the activation energy for conduction. As summarized in Table 3, *E_a_* values were close to the reported activation energy for the single- and doubly-ionized oxygen vacancies, which are typically in the 0.1–0.5 and 0.6–1.2 eV ranges, respectively [38]. Moreover, the value of the activation energy of grain conduction increases slightly by cosubstitution. In contrast, the value of *E_a_* of grain-boundary conduction considerably decreases for cosubstituted ceramics.

Oxygen vacancies develop during a high-temperature (>1000 °C) sintering step due to oxygen loss [39]. Additional free electrons are expected by the partial substitution of Ti^4+^ by the pentavalent Nb^5+^, which explains the reduced resistivity of CCTANO and CCTBNO ceramics as compared to the pure CCTO sample. Moreover, the substitution of the Bi^3+^ acceptor ions for Ti^4+^ requires oxygen vacancies for charge conservation. Possible reaction equations, written using the Kröger–Vink notation for defects, are as follows:

(5) $$O_{o}\Leftrightarrow \frac{1}{2}O_{2}+V_{o}^{\prime \prime }+2e^{-}$$

(6) $$2Nb_{2}O_{5}\Leftrightarrow 4Nb_{Ti}^{•}+8O_{o}+O_{2}\left(g\right)+4e^{-}$$

(7) $$Bi_{2}O_{3}\Leftrightarrow 2Bi_{Ti}^{\prime }+V_{o}^{••}+3O_{o}.$$

**Figure 7:**
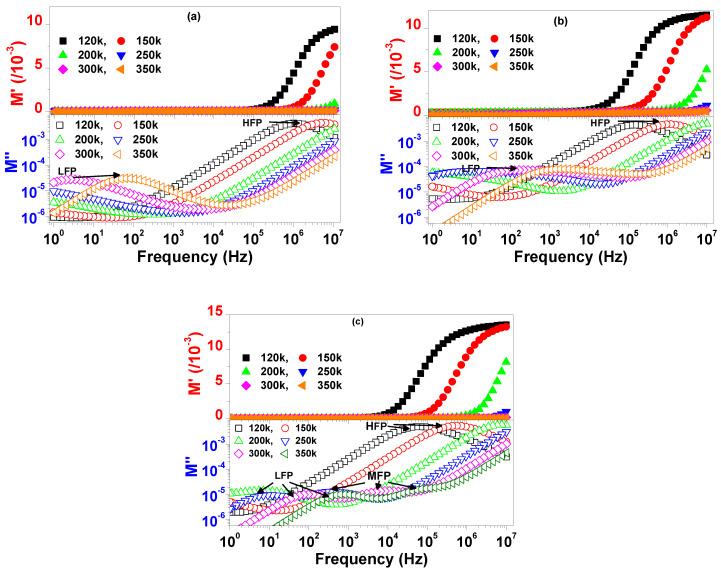
Spectra of M’ and M’’ for (**a**) CCTO, (**b**) CCTBNO, and (**c**) CCTANO ceramics at selected temperatures.

Considering the findings of complex-impedance measurements, the effect of Nb^5+^ dopants in reducing resistivity was more pronounced for grain boundaries, which explains the comparatively higher tanδ of the cosubstituted ceramics compared with that of CCTO. Moreover, free electrons might have been captured by Cu^2+^ and Ti^4+^, thus forming Cu^+^ and Ti^3+^ ions in order to maintain charge balance. Thus, the hopping of electrons between oxygen vacancies and mixed-valent structure of Cu+/Cu^2+^ and Ti^3+^/Ti^4+^ might have contributed to conduction in the present ceramics. Figure 7a–c show the spectra of the real (M’) and imaginary (M’’) parts of the electric modulus at selected temperatures of 120, 150, 200, 250, 300, and 350 K. M’ values were nearly zero at low frequencies for all samples and increased with frequency, which indicated the suppression of electrode polarization effects [40]. Two relaxation peaks could be seen in the spectrum of M’’ for CCTO and CCTBNO. At low temperatures, the first peak appeared at a high frequency (HFP); then, with increasing temperature, a second peak began at low frequencies (LFP). Both peaks were thermally activated, where they shifted towards higher frequencies with increasing temperature. The existence of high- and low-frequency peaks is commonly reported for CCTO-based ceramics, and is attributed to the response of the grain and grain boundary, respectively [12,30]. 

**Figure 8:**
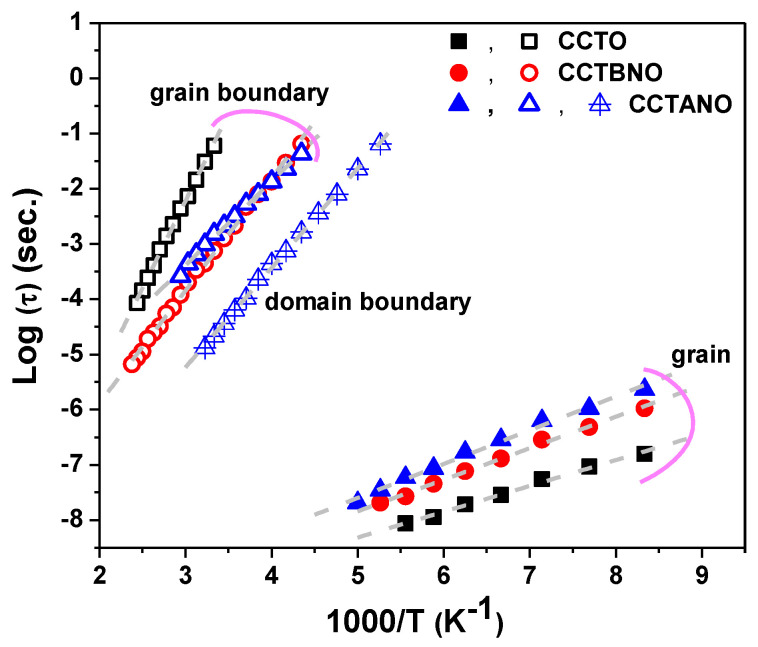
Arrhenius plots of relaxation time for CCTO, CCTANO, and CCTBNO ceramics.

As depicted in Figure 7c, three thermally activated peaks existed in the spectra of M’’ for the CCTANO sample. In addition to the commonly reported low- and high-frequency peaks, a third peak appeared at medium frequencies (MFP). The frequency at the peak maximum of the M’’ spectra (f_max_) was related to relaxation time (τ) as τ = 1/2πf_max_. As shown in Figure 8, temperature dependency of τ follows the Arrhenius law (Equation (3)). Calculated *E_R_* values related to the different relaxation peaks are given in Table 3. *E_R_* values were close to the *E_a_* values, and both were in the reported range of activation energies of oxygen vacancies, as previously discussed. Thus, the single- and doubly-ionized oxygen vacancies played a considerable role in the relaxation and conduction responses of the investigated samples.

## 4. Conclusions

CaCu_3_Ti_4-x_[(A_0.05_Nb_0.05_)]_x_O_12_ ceramics (A: Al and Bi; x = 0, 0.3) were synthesized by high-energy mechanical ball milling and reactive sintering inside a tubular furnace for 15 h at 1050 °C in air. X-ray diffraction measurements revealed a cubic CCTO structure as the main phase for all ceramic samples. Nevertheless, the (Bi^3+^, Nb^5+^)-cosubstituted CCTO demonstrated a secondary pyrochlore phase content of ~18% (mole ratio). It also showed the smallest and most uniform grain size of ~3 μm compared to the pure (7–9 μm) and (Al^3+^, Nb^5+^)-cosubstituted CCTO (6–12 μm). The studied ceramics showed colossal relative permittivity (*ε’* > 10^3^) over wide ranges of temperature and frequency, with the (Al^3+^, Nb^5+^)-cosubstituted CCTO sample having the highest relative permittivity of *ε’* = 3.9 × 10^4^ at 1 kHz and 300 K. The giant relative permittivity of the ceramics could be attributed to the Maxwell–Wagner polarization effect at the blocking grain and domain boundaries. Meanwhile, cosubstituted CCTO samples of the present study revealed degradation of its dielectric loss (tanδ), which was attributed to the decrease in grain-boundary resistance, as revealed by impedance-spectroscopy analysis. Modulus-spectrum analysis revealed two relaxation processes for the pure and (Bi^3+^, Nb^5+^)-cosubstituted CCTO samples. Dissimilar behavior was observed for (Al^3+^, Nb^5+^)-cosubstituted CCTO, where three relaxation mechanisms contributed and were attributed for the grain, domain-boundary, and grain-boundary responses. The calculated activation energies highlighted the role of single- and doubly-ionized oxygen vacancies in the relaxation and conduction responses of the investigated samples. 

## Figures and Tables

**Table 1:** Lattice parameter (a), unit cell volume (V) for main CCTO phase, and agreement factors of profile (Rp), weighted profile (Rwp), structure (RF), and goodness of fit (GofF) obtained through Rietveld refinement.

Sample	CCTO	CCTANO	CCTBNO
Space group	Im3	Im3	Im3
a (Å)	7.394 (3)	7.397 (8)	7.40056
V (Å^3^)	404.28 (1)	404.86 (1)	405.3165
R_p_ (%)	11.820	16.316	11.256
R_wp_ (%)	15.552	21.429	15.357
R_B_ (%)	10.453	13.805	7.741
R_F_ (%)	8.582	12.305	5.675
GofF	1.74	2.22	2.0

**Table 2:** Values of equivalent-circuit parameters at selected temperatures by ZSimpWin fitting.

Sample	Temperature/Equivalent Circuit	R_1-grain_ Ω.cm	CPE_1_		R_2-*g.b.*_ Ω.cm	CPE_2_		R_3-*d.b.*_ Ω.cm	CPE_3_	
			C_1_ (nF)	n_1_		C_2_ (nF)	n		C_3_ (nF)	n_3_
CCTO	200 K 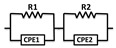	470	0.016	0.99	3.4 × 10^9^	2.6	0.98	-	-	-
	300 K 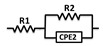	63	-	-	48 × 10^6^	31.3	0.92	-	-	-
CCTBNO	200 K 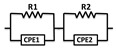	573	0.029	0.99	3.8 × 10^8^	1.3	0.96	-	-	-
	300 K 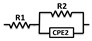	191	-	-	4.3 × 10^6^	1.1	0.91	-	-	-
CCTANO	200 K 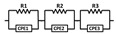	3592	0.024	0.93	24 × 10^7^	13.6	0.98	2 × 10^6^	10	0.96
	300 K 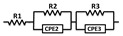	189	-	-	1.4 × 10^5^	6.1	0.93	1.8 × 10^3^	13	0.95

**Table 3:** Activation energy for conduction *E_a_* (eV) and relaxation *E_R_* (eV).

Sample	*E_a_*_(*g.*)_	*E_a_*_(*d.b.*)_	*E_a_*_(*g.b.*)_	*E_R_*_(*PHF*)_	*E_R_*_(*PMF*)_	*E_R_*_(*PLF*)_
CCTO	0.093	-	0.628	0.099	-	0.751
CCTBNO	0.113	-	0.404	0.114	-	0.444
CCTANO	0.121	0.357	0.301	0.131	0.390	0.327
